# B cell repertoires in HLA-sensitized kidney transplant candidates undergoing desensitization therapy

**DOI:** 10.1186/s12967-017-1118-7

**Published:** 2017-01-13

**Authors:** John F. Beausang, H. Christina Fan, Rene Sit, Maria U. Hutchins, Kshama Jirage, Rachael Curtis, Edward Hutchins, Stephen R. Quake, Julie M. Yabu

**Affiliations:** 1 CareDx, 3260 Bayshore Blvd, Brisbane, CA 94005 USA; 2 Immumetrix, LLC, 3183 Porter Drive, Palo Alto, CA 94304 USA; 3 Department of Bioengineering, Stanford University, 318 Campus Drive, Stanford, CA 94305 USA; 4 Howard Hughes Medical Institute, Stanford, CA USA; 5 Department of Medicine, Stanford University School of Medicine, 750 Welch Road, Palo Alto, CA 94304 USA

**Keywords:** Kidney transplantation, HLA sensitization, B cells, Immune repertoire, DNA sequencing, Desensitization

## Abstract

**Background:**

Kidney transplantation is the most effective treatment for end-stage renal disease. Sensitization refers to pre-existing antibodies against human leukocyte antigen (HLA) protein and remains a major barrier to successful transplantation. Despite implementation of desensitization strategies, many candidates fail to respond. Our objective was to determine whether measuring B cell repertoires could differentiate candidates that respond to desensitization therapy.

**Methods:**

We developed an assay based on high-throughput DNA sequencing of the variable domain of the heavy chain of immunoglobulin genes to measure changes in B cell repertoires in 19 highly HLA-sensitized kidney transplant candidates undergoing desensitization and 7 controls with low to moderate HLA sensitization levels. Responders to desensitization had a decrease of 5% points or greater in cumulated calculated panel reactive antibody (cPRA) levels, and non-responders had no decrease in cPRA.

**Results:**

Dominant B cell clones were not observed in highly sensitized candidates, suggesting that the B cells responsible for sensitization are either not present in peripheral blood or present at comparable levels to other circulating B cells. Candidates that responded to desensitization therapy had pre-treatment repertoires composed of a larger fraction of class-switched (IgG and IgA) isotypes compared to non-responding candidates. After B cell depleting therapy, the proportion of switched isotypes increased and the mutation frequencies of the remaining non-switched isotypes (IgM and IgD) increased in both responders and non-responders, perhaps representing a shift in the repertoire towards memory B cells or plasmablasts. Conversely, after transplantation, non-switched isotypes with fewer mutations increased, suggesting a shift in the repertoire towards naïve B cells.

**Conclusions:**

Relative abundance of different B cell isotypes is strongly perturbed by desensitization therapy and transplantation, potentially reflecting changes in the relative abundance of memory and naïve B cell compartments. Candidates that responded to therapy experienced similar changes to those that did not respond. Further studies are required to understand differences between these two groups of highly sensitized kidney transplant candidates.

**Electronic supplementary material:**

The online version of this article (doi:10.1186/s12967-017-1118-7) contains supplementary material, which is available to authorized users.

## Background

Kidney transplantation is the most effective form of therapy for end-stage renal disease (ESRD) in terms of mortality, quality of life and health care savings [1]. Human leukocyte antigen (HLA) sensitization is a major barrier to successful kidney transplantation, especially amongst the highly sensitized. Sensitized kidney transplant candidates comprise approximately 30% of the deceased donor waiting list and have the longest wait times because of difficulty in finding a compatible donor [2]. HLA sensitization refers to pre-existing antibodies against human HLA proteins that are produced in the transplant candidate after contact with non-self HLA antigens, commonly from previous transplants, pregnancies and blood transfusions [3–5]. Antigen-specific B cells recognize, bind, internalize and process the antigens through the B cell receptor (BCR) [6]. When a co-stimulatory signal is present from CD4^+^ T cells, B cells undergo clonal expansion, producing plasmablasts, which secrete high affinity HLA antibodies, and memory B cells, which can provide a source of HLA antibodies long after the sensitizing event [6].

Desensitization therapies include plasmapheresis, which physically removes proteins from sera, intravenous immunoglobulin (IVIG), which can decrease circulating HLA antibody proteins, anti-CD20 monoclonal antibody treatment (rituximab), which depletes most B cells with the notable exception of antibody-producing plasma cells, and proteasome inhibitors (bortezomib), which target plasma cells. Candidates who respond to desensitization therapy with a decrease in HLA antibodies and undergo successful transplantation show a survival benefit compared to remaining on dialysis [7, 8]. However, for unknown reasons, circulating HLA antibody levels do not decrease in a significant number of sensitized kidney transplant candidates following desensitization therapy, and potential toxicity from medications could lead to unwarranted risk and poor outcomes. The degree of sensitization to individual donor HLA proteins is measured using single antigen bead assays. Defining HLA matching at the epitope level may allow for a more detailed assessment of HLA compatibility by addressing the immunogenicity of a particular HLA antigen mismatch [9]. This information is crucial for matching donated organs with transplant candidates, but it is not informative as to which sensitized candidates will respond to desensitization therapy [10, 11].

Genomic rearrangement of variable (V), diversity (D) and joining (J) segments of immunoglobulin genes generates a diverse set of BCRs that recognize different antigens [12, 13]. Most of the diversity in the antibody repertoire is in the hypervariable complementarity-determining region 3 (CDR3) of the heavy chain of the BCR and largely accounts for antibody specificity [14]. The constant region determines the antibody isotype. Naïve B cells produce both IgM and IgD isotypes. After encountering antigen, B cells undergo clonal expansion and somatic hypermutation, affinity maturation, and class-switch recombination to produce IgG, IgA or IgE antibodies [15]. High-throughput DNA sequencing of immunoglobulin genes has been used to detect and follow residual disease in B cell malignancies, determine vaccine response, and perhaps has a role in autoimmune disease diagnostics [16–20]. We reported that repertoire sequencing is correlated with immune activity during rejection episodes in heart transplant recipients [21].

Investigations of HLA sensitization may benefit from B cell immune repertoire sequencing because the antigen is known and several therapies that target B cells and/or decrease HLA antibodies are available. We developed an assay utilizing high-throughput DNA sequencing of the variable domain of the antibody heavy chain of immunoglobulin genes to measure changes in B cell repertoires before and after desensitization therapy and transplantation. Our objective was to survey a small cohort of highly sensitized kidney transplant candidates to determine whether measuring baseline and longitudinal B cell repertoires could serve as early biomarkers to help select candidates that will respond to therapy.

## Methods

### Kidney transplant candidates

**Fig. 1:**
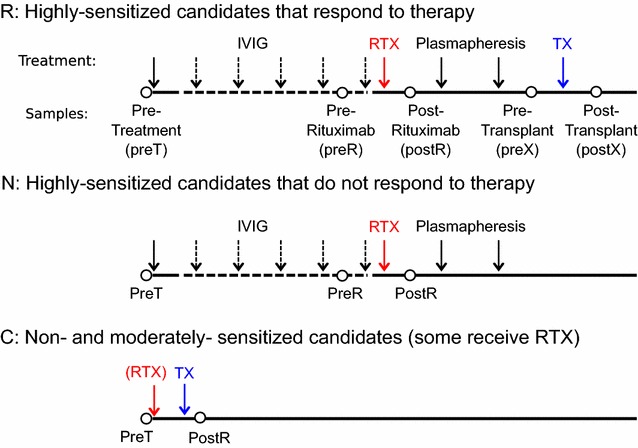
Candidate groups and schematic for desensitization protocol

19 highly HLA-sensitized candidates with ESRD and cumulative calculated panel reactive antibody (cPRA) scores of 93–100% were treated with desensitization therapy. Eleven candidates responded to therapy and received transplants (“responders”) whereas eight did not respond to therapy (“non-responders”). After more than 6 months of desensitization therapy without a decrease in HLA antibodies, three non-responders (N-03, N-05 and N-06) received well-matched transplants. They remained non-responders in our analysis based on lack of a response to therapy. Candidates were enrolled in the protocol based on waiting time on the deceased donor kidney transplant list or availability of an incompatible living donor. Seven additional candidates with ESRD and cPRA scores of 0–41% who received transplants served as controls (Fig. 1). Age, sex, race, cause of ESRD, cPRA values before and after therapy, response to therapy and transplantation status are summarized in Table 1 and Additional file 1: Table S1. Written informed consent was obtained from all candidates. The consent included HIPPAA authorization for access to medical records. The Institutional Review Board at Stanford University approved the protocol (15267 and 17997).

**Table 1:** Candidate demographics, cPRA and clinical outcomes

	Controls (n = 7)	Responders (n = 11)	Non-responders (n = 8)
Age (years)	45 ± 11	40 ± 12	43 ± 7
Male/female	4/3	7/4	2/6
Race			
White	3	3	4
Black	1	3	1
Hispanic	1	1	3
Asian	2	3	0
Native American	0	1	0
Cause of ESRD			
Diabetes	0	2	1
Glomerulonephritis	4	1	1
SLE	1	3	1
PKD	0	1	1
Congenital	0	3	0
Other	2	1	4
Cumulative cPRA (%)			
Before desensitization	13 ± 16	97.4 ± 2.7	100 ± 0
After desensitization	N/A	87.9 ± 3.8	100 ± 0
Transplanted (%)	7 (100)	11 (100)	3 (37.5)
Donor type: DD:LUR:LRD	1:0:6	8:2:1	2:1:0
Rejection			
Borderline acute	0	3 (27)	1 (33)
Cell mediated	0	1 (9)	0
Antibody mediated	0	0	0
Graft loss (%)	0	0	0
Death-censored graft survival (1 year)	100%	100%	100%

*SLE* systemic lupus erythematosus, *PKD* polycystic kidney disease, *DD* deceased donor, *LUR* living unrelated, *LRD* living related donor

### HLA antibody measurements

cPRA is the traditional measurement for the degree of sensitization in transplant candidates [2, 22]. cPRA is an index of the probability of finding a compatible donor and is based on HLA antibody specificities and strength detected in the serum combined with the frequency of those HLA antigens in the donor population. Therefore, cPRA estimates the percentage of donors with whom a candidate would be incompatible with a score of 0% indicating no existing HLA antibodies and a good chance of receiving a matched organ and a score of 100% indicating multiple, high strength antibodies to common HLA alleles and thus almost no chance of finding a match. Candidates with a cPRA score of 80% or more are considered highly sensitized [23]. Luminex HLA Class I and II single antigen beads and Fusion analysis (LabScreen, One Lambda, Canoga Park, CA, USA) were used to determine HLA antibody specificities. HLA antibodies with ≥1000 normalized mean fluorescence intensity (MFI) was considered positive. cPRA was determined using HLA frequencies from United Network for Organ Sharing (UNOS) based on a recent cohort of deceased donors.

### Desensitization protocol and outcome measures

A modified version of the high-dose IVIG and rituximab protocol previously reported by Vo and Jordan was used [23]. All candidates were treated with monthly IVIG at 2 g/kg, maximum dose 140 g, prior to single-dose rituximab 375 mg/m^2^ intravenously administered either after the first or sixth dose of IVIG. Re-dosing of rituximab was based on measurement of any detectable B cells by quantitative flow cytometry on peripheral blood. One candidate (C-07) received a second dose of rituximab based on the blood incompatible protocol. If there was no change in cPRA after 6–12 months of IVIG and rituximab, candidates were treated with bortezomib 1.3 mg/m^2^ intravenously every 72 h for four doses and plasmapheresis for 1–2 cycles following a modified protocol previously reported by Woodle [24].

Response to therapy was assessed by a decrease of 5% points or greater in cPRA. This threshold was based on published clinical studies and transplant data that concluded a 5% point decrease in cPRA is clinically relevant and corresponds to a significant decrease in HLA antibody strength to enable transplantation with a compatible donor by increasing the potential donor pool [2, 25, 26]. In our study, this decrease in cPRA corresponded to a decrease in the strength of two or more HLA antibodies in responders. Non-response was defined as no decrease in HLA antibodies as measured by cPRA after all desensitization therapies were completed. Candidates who received transplants were treated with anti-thymocyte globulin (ATG) induction therapy and mycophenolate mofetil (MMF), tacrolimus and prednisone for maintenance immunosuppression.

### Sample collection and processing

Serial samples of peripheral blood were collected to follow B cell immune repertoires pre-treatment, after desensitization therapy and after transplantation. Samples from controls were previously obtained. Peripheral blood mononuclear cells (PBMC) for immunoglobulin sequencing were isolated by Ficoll gradient centrifugation and cryopreserved in the Human Immune Monitoring Center (HIMC) at Stanford University as previously described [27]. RNA was extracted using a Qiagen AllPrep Kit and quantitated using Bioanalyzer and Quibit (Additional file 1: Table S2).

### Immunoglobulin sequencing

Immunoglobulin RNA molecules were converted into Illumina sequencing libraries using a 5′RACE based assay with reverse transcription primed from one of five isotypes (IgD, IgM, IgA, IgG or IgE) constant regions and template-switching enzyme (Superscript II, Invitrogen 18064-014) that incorporates an oligo containing a random 9-mer molecular tag and partial Illumina adapter to the 5′ end of each cDNA molecule (ImmuMetrix, LLC; Additional file 1: Table S3, Additional file 2). Subsequent rounds of PCR amplification incorporate full sequencing adaptors, including a barcode specific to the sample for de-multiplexing pooled sequencing data. Libraries from 81 samples were constructed and sequenced on an Illumina MiSeq instrument with 2 × 150 bp chemistry, yielding a median sequencing depth of ~70 k B cell reads per sample (Additional file 1: Table S2 for details).

### Data analysis

We analyzed V gene usage, mutation rates, abundance and CDR3 lengths in candidates undergoing desensitization therapy and transplantation. Sequenced reads were passed through a custom analysis pipeline in order to identify isotype composition, gene segment usage and point mutations from germline. First, reads were segregated by identical 9 mer barcodes, which were then clustered to distinguish different molecules tagged with the same barcode. PCR duplicates and sequencing errors were removed by constructing a consensus sequence within each cluster using either the majority base or the base with the highest quality score when only two reads were present. The raw sequence was used for clusters containing a single read. These high-quality sequences were then aligned using Smith–Waterman sequence alignment to the constant region and V, D and J germline gene segments as defined in the IMGT immunoglobulin database [28]. Somatic hypermutation was estimated by dividing the total number of mismatches (relative to the corresponding IMGT reference sequences) in the leader sequence and partially sequenced V region by the length of these regions sequenced (Additional file 2). In order to correct for small biases in the mutation frequency between sequencing runs, the average mutation frequency across all samples in a sequencing run was subtracted from the average across all lanes. This difference was then applied as a correction factor to each sample in that lane (see Additional file 1: Table S4 for details).

Features of the immune repertoire, including V gene usage, relative isotype distribution and V gene region mutation frequency, were determined using programs in C++, bash and R. Sequence abundance, isotype composition and mutation frequency were reported after subsampling the data to 1000 molecules, and significance of the results were confirmed at multiple depths including no subsampling. Average isotype abundance and mutation frequency were weighted by the number of molecules in each of the four isotypes IgG, IgA, IgM and IgD. Switched isotypes refer to IgG and IgA and non-switched isotypes to IgM and IgD. The fraction of IgE molecules was low (<1% of molecules) in all samples and was omitted from the analysis. Changes in number of sequences, isotype distribution and mutation frequency after IVIG, rituximab and transplantation were determined by subtracting the frequency determined in the sample following therapy from the frequency prior to therapy. V gene usage in pre-treatment samples from the three groups (responders, non-responders and controls) was determined by averaging the molecule abundance of each V gene from all the candidates. The relative usage of different V-J gene combinations (44 V genes × 6 J genes = 264) was compared by determining the relative fraction of molecules in each combination and calculating the correlation coefficient between all pairs of candidates.

Significance tests to assess changes after treatment were performed in R using the paired Wilcoxon–Mann–Whitney test. For pre-treatment comparisons among the three groups, significance was determined using Kruskal–Wallis one-way analysis of variance test. p values less than 0.05 were confirmed by bootstrapping with 1000 iterations of the test applied to random samples drawn from the data. To determine which of the three groups is significantly different from the others, the Nemenyi test was applied (kruskalmc function in R) and comparisons with p value <0.05 were reported.

Samples from three groups (responders, non-responders and controls) were evaluated in four phases: (1) pre-treatment, (2) after IVIG, (3) after rituximab and (4) after transplantation. The samples collected from each candidate and relative timing of therapies and transplantation are detailed in Fig. 1 and Additional file 1: Table S2. For the pre-treatment analysis, 21 candidates from all three groups had baseline samples available. To assess the effect of IVIG alone, nine candidates had samples available before and after IVIG alone (dashed line in Fig. 1). In candidates receiving IVIG, the sample prior to rituximab was compared to the pre-treatment sample. To assess the effect of rituximab, 19 candidates who had samples before and after rituximab were included. Included in this analysis were two candidates (R-04 and R-09) who received a single dose of bortezomib within 2 weeks of receiving rituximab and two controls (C-01 and C-06) who received a single dose of rituximab as part of a blood group incompatible protocol. The effects of transplantation were evaluated in 10 of 13 candidates receiving a transplant. Three candidates (C-05, C-06 and C-07) were excluded due to receiving rituximab within 30 days prior to transplantation; samples from two of these candidates (C-06 and C-07) contributed to other analyses. We performed the post-transplant analysis with the three candidates included and found no significant differences in our results. In addition to these 13 candidates, six responders and two non-responders received transplants after sample collection was completed (see Additional file 1: Table S2).

## Results

### Candidate characteristics

Demographic, clinical characteristics, cPRA and clinical outcomes of the candidates are summarized in Table 1. Eleven responders had a decrease in cPRA (range 5–13% with mean 9.5 ± 2.3%) and received transplants after desensitization therapy. Three non-responders received transplants from HLA-matched donors, and five failed to receive transplants.

### Baseline B cell repertoires

**Fig. 2:**
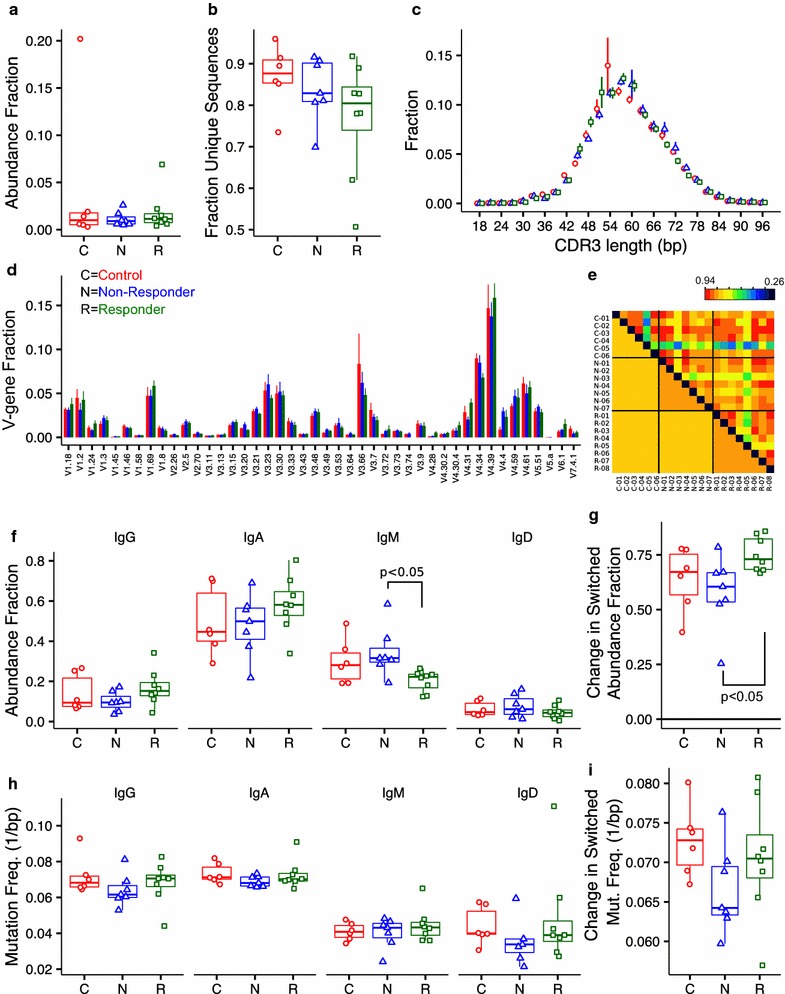
Baseline B cell repertoires. Baseline B cell repertoires in six control, seven non-responding and eight responding candidates prior to receiving desensitization therapy. **a** The fraction of molecules corresponding to the most abundant sequence. **b** The fraction of unique sequences detected after subsampling to 1000 molecules in each sample. **c** Histogram of in-frame CDR3 lengths averaged over the samples in each group. **d** V-gene usage averaged across samples in each group. **e** Cross correlation matrix of the abundances in all V-J gene combinations between all samples (individual elements *above the diagonal*) and averaged within each group (large regions *below diagonal*). **f** Molecule-weighted isotype abundance in each sample. **g** Fraction of molecules in each sample containing a switched isotype (i.e., sum of IgG and IgA from **d**). **h** Molecule-weighted mutation frequency for each isotype. **i** Mutation frequency averaged across isotypes for each sample (data from **f**)

To determine if highly sensitized candidates had a dominant circulating B cell clone that may be responsible for producing HLA antibodies, we compared the abundance of the top clone in pre-treatment samples from six controls (C), seven non-responding (N) and eight responding (R) candidates (Fig. 2a). Most samples regardless of sensitization status contained similar distributions with maximum clone sizes of <3% except for two outliers containing dominant clones of 20% and 6.9% in C and R groups, respectively. After subsampling to 1000 molecules, the fraction of unique sequences was not significantly different between the three groups, ranging between 0.70 and 0.92 for 18 out of 21 samples (Fig. 2b). The two lowest fractions in the R group were from samples with the two lowest numbers of input molecules, arguing against any potential trend for lower sequence diversity in non-responders and responders relative to controls. The distribution of CDR3 lengths (Fig. 2c) was similar in the three groups with nearly identical average lengths (58.3–59.3 bp) and standard deviations (10.0–10.3 bp). The average V gene usage was not significantly different between the three groups (Fig. 2d). A more detailed comparison based on the cross-correlation of the different V-J gene abundances in each sample also did not distinguish any differences when visualized individually (elements above the diagonal in Fig. 2e) or averaged within each group (elements below the diagonal in Fig. 2e).

The relative isotype abundance for each sample (Fig. 2f) was similar for the three groups with elevated IgA and IgM (median fractions of 0.54 and 0.26, respectively) and lower IgG and IgD (median fractions of 0.11 and 0.05, respectively). Candidates responding to therapy had significantly lower fractions of IgM (p < 0.05) compared to non-responders and a trend towards lower IgD and higher IgA and IgG (Fig. 2f). To further confirm this finding, we combined switched isotypes (IgG and IgA) and detected a small increase in the fraction of the combined switched isotypes from 0.67 to 0.73 in responders compared to non-responders (p < 0.05; Fig. 2g). The fraction of switched isotypes in controls was not significantly different from either responders or non-responders (Fig. 2g). V region mutation frequencies were similar in the three groups with IgG and IgA approximately 0.07 mismatches per base compared with 0.045 mismatches per base for IgM and IgD (Fig. 2h). Averaging the mutation frequency across the combined switched isotypes did not show any significant differences (Fig. 2i).

Peripheral B cell percentages at baseline were sufficient and not significantly different between responders and non-responders (mean B cell percentage of total lymphocytes 6.8 ± 3.8%) and were also similar to B cell percentages in healthy controls as shown in a previous analysis [29].

### B cell repertoires after IVIG

**Fig. 3:**
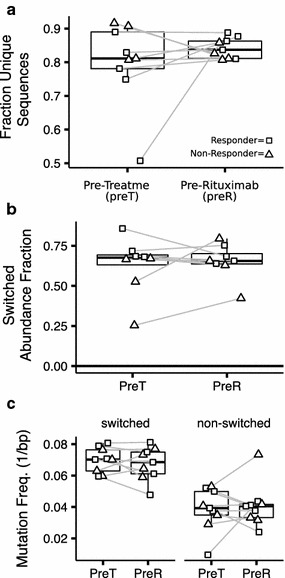
B cell repertoires after IVIG. B cell repertoires after IVIG in four non-responding and five responding candidates following the pre-treatment sample and ending with the sample immediately prior to receiving rituximab. **a** The fraction of unique sequences detected after subsampling to 1000 molecules. **b** The relative abundance of switched isotypes. **c** Switched and non-switched V-gene mutation frequency. Samples from the same candidate are connected by a *gray line*

Nine out of 19 highly sensitized candidates (five responders and four non-responders) had samples available before and after receiving IVIG alone. The median fraction of unique sequences of 0.81 at the pre-treatment time point was not significantly different from 0.84 after IVIG (Fig. 3a). The median abundance of molecules containing switched isotypes was constant at 0.67 before and after IVIG (Fig. 3b). The median mutation frequency in switched and non-switched molecules was also unchanged at approximately 0.07 and 0.04 mismatches per base, respectively (Fig. 3c). See Additional file 3: Figure S1a, b for individual isotype abundance and mutation frequencies.

### B cell repertoires after rituximab

**Fig. 4:**
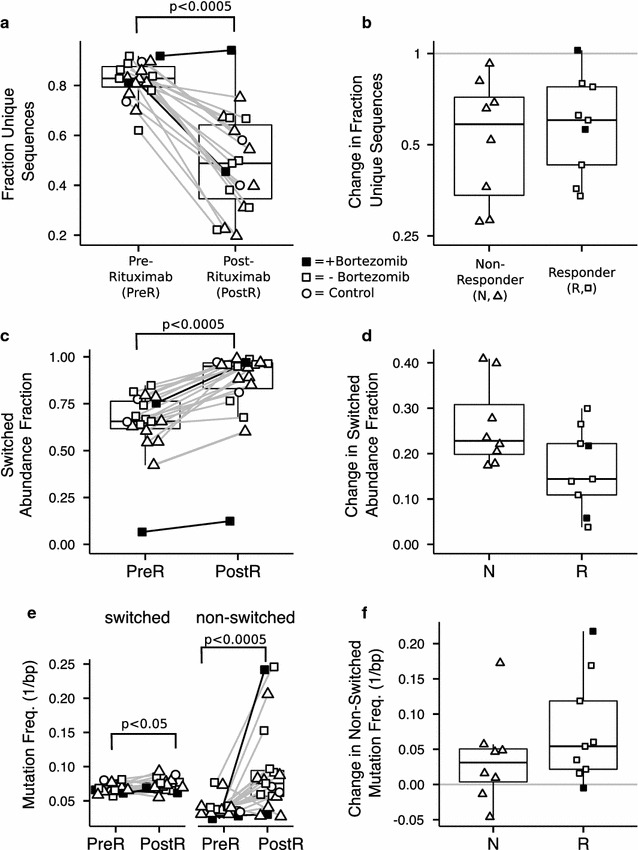
B cell repertoires after rituximab. B cell repertoires after rituximab in two controls, eight non-responders (N) and nine responders (R) before and after rituximab. **a** The fraction unique sequences detected in each sample after subsampling to 1000 molecules. **b** The change in the fraction of unique sequence in the N and R groups. **c** The relative abundance of molecules with switched isotypes. **d** The change in abundance of the N and R groups. **e** Mutation frequency for switched and non-switched isotypes. **f** The change in non-switched mutation frequency for N and R groups. Samples from the same candidate are connected by a *line*

Nineteen out of 26 candidates (eight non-responders, nine responders and two controls) had samples available before and after receiving rituximab. Rituximab reduced the median fraction of unique sequences by 40% across all candidates with only one candidate experiencing a small increase (p < 0.0005; Fig. 4a). The magnitude of the decrease was not significantly different between responders and non-responders (Fig. 4b). After rituximab, the fraction of switched isotypes increased significantly from a median of 0.65–0.95 (p < 0.0005; Fig. 4c). There was a trend towards a smaller increase in switched isotypes in responders (median increase 0.14) compared to non-responders (median increase 0.23); however, the difference was not significant (p = 0.09; Fig. 4d). The mutation frequency of switched isotypes increased in 14 out of 19 candidates with a small increase in the median from 0.065 to 0.070 mismatches per base (p < 0.05; Fig. 4e). However, the mutation frequency of non-switched isotypes increased by nearly twofold from 0.038 to 0.075 mismatches per base (p < 0.0005; Fig. 4e). There was a trend towards a larger increase in the non-switched mutation frequencies in responders (median increase 0.031) compared to non-responders (median increase 0.054) but the difference was not significant (Fig. 4f).

Immediately after rituximab, peripheral B cells were undetectable in all candidates. Approximately half of post-rituximab samples were collected within 31 days of therapy, and all but one sample from the other half were collected within 54 days. Comparing these two groups, there was a small but not statistically significant increase in the magnitude of the change in isotype abundance and class-switched mutation frequency in samples collected more than 31 days after transplantation (see Additional file 4: Figure S4a, b).

Two candidates also received bortezomib during the same period as rituximab (black symbols in Fig. 4a; Additional file 1: Table S2). One candidate (R-04) showed a small increase in unique sequences, and the other candidate (R-09) showed a decrease in unique sequences similar to the majority of the candidates (Fig. 4a). Prior to receiving bortezomib, R-04 was an outlier with a low abundance of switched molecules (0.07) relative to the other candidates (see Additional file 3: Figure S1c, d). However, despite this lower initial abundance of switched isotypes, the fraction of unique sequences increased after rituximab consistent with the other candidates (Fig. 4a).

### B cell repertoires after kidney transplantation

**Fig. 5:**
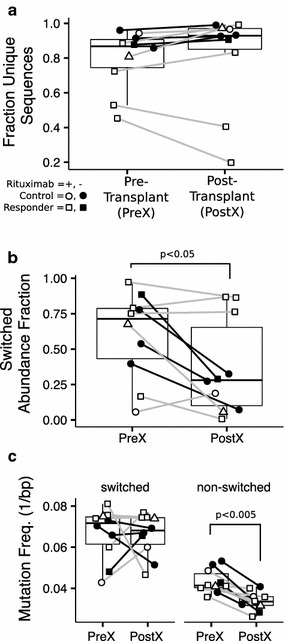
B cell repertoires after transplantation. B cell repertoires after transplantation in four controls and five responding candidates before and after transplantation. **a** The number of unique sequences after subsampling to 1000 molecules. **b** The fraction of molecules corresponding to switched isotypes. **c** The mutation frequency in switched and non-switched isotypes. Samples from the same candidate are connected by a *line*

Ten candidates (four controls, five responders and one non-responder) had samples available shortly before and after kidney transplantation. Six of the ten candidates received rituximab more than 30 days prior to receiving the transplant whereas the remaining four did not receive rituximab. The fraction of unique sequences increased in eight out of ten candidates from a median fraction of 0.87 before transplant to 0.93 after transplant but was not significant (Fig. 5a). The two candidates (R-01 and R-11) who showed a decrease in unique sequences also had the lowest fraction of unique sequences prior to transplantation (0.53 and 0.43, respectively). After transplantation, the change in abundance of individual isotypes showed significant increases in IgM and IgD (p < 0.05), a decrease in IgA (p < 0.05), and a trend towards decreasing IgG (p = 0.10; Additional file 3: Figure S1e, f). Consistent with these observations, the switched isotype abundance decreased from a median of 0.71 to 0.28 after transplantation (p < 0.05; Fig. 5b). The mutation frequency of switched isotypes (~0.07 mismatches per base) was not significantly different after transplantation, whereas the mutation frequency of non-switched isotypes decreased from 0.41 to 0.33 mismatches per base (p < 0.005; Fig. 5c).

In post-transplant samples from our previous analysis, there was repopulation of B cells to levels close to baseline, which is consistent with findings from other studies [29, 30]. Post-transplant samples were collected between five and 70 days following transplantation. There was no significant impact in the change in isotype abundance or mutation frequency based on sampling interval although two of the three candidates with increased class-switched isotype abundance following transplantation were collected approximately 10 days earlier than the other samples, suggesting that these outliers may partly be explained by sampling at an earlier time after transplantation. All candidates show a decrease in non-switched mutation frequency after transplantation regardless of sampling interval (see Additional file 4: Figure S4c, d).

## Discussion

In this study we developed a high-throughput DNA sequencing assay to measure circulating B cell repertoires in highly HLA-sensitized kidney transplant candidates undergoing desensitization therapy to lower HLA antibodies and enable transplantation. We hypothesized that measuring circulating B cell repertoires could help identify which candidates will respond to desensitization therapy. cPRA is a measure of sensitization based on pre-existing HLA antibody specificities and strength in combination with the frequency of HLA antigens in the donor population. Since cPRA also represents the probability of receiving a compatible transplant, this measurement is often used to determine success of desensitization therapy [23, 31]. We assessed “response” to therapy as a clinically meaningful and durable decrease in cPRA of 5% points or greater based on literature and transplant outcomes data [2, 26].

We first compared B cell repertoires in pre-treatment samples in three groups: sensitized candidates who responded to desensitization therapy, sensitized candidates who did not respond to desensitization therapy, and control candidates with low to moderate levels of HLA sensitization. Our results do not identify a single dominant B cell clone circulating in highly sensitized candidates compared to controls, but we cannot rule out the presence of one or more smaller clones that may be responsible for producing HLA antibodies. The analysis of several different metrics including the fraction of unique sequences, CDR3 length, V gene and V-J gene abundances, and isotype mutation frequencies argues against a common feature of the repertoire that predisposes candidates to high levels of HLA antibody production. Possible explanations are that the B cell clones circulate at low levels in the peripheral blood or the presence of more dominant B cells makes it difficult to detect by our assay. It has been suggested that no more than 2% of all B cells are present in the peripheral blood compared to the lymph nodes, spleen and bone marrow [32], and the relevant HLA-specific B cell clones may be sequestered in another compartment and only rarely present in circulation.

Before desensitization therapy, responders had a slightly higher fraction of switched (IgG and IgA) isotypes compared to non-responders, but without a corresponding increase in mutation frequency. This shift may represent more memory B cells or plasmablasts in the circulation prior to therapy although the relatively small magnitude of the change would need to be validated in a larger study. Memory B cells are thought to be important in HLA sensitization as they can quickly transform into antibody-secreting plasma cells upon re-exposure to alloantigen [33]. Thus, depleting these circulating memory B cells may reduce the number of plasma cells, and ultimately, the production of high affinity HLA antibodies.

We next analyzed changes in the B cell repertoires after desensitization therapy. After multiple doses of IVIG, we did not detect any changes in the B cell repertoire. IVIG is purified immunoglobulin products derived from pooled human plasma from thousands of donors and typically contains more than 95% unmodified IgG and trace amounts of IgA or IgM [34]. IVIG has been used in transplantation to decrease HLA antibodies in order to help enable transplantation in highly sensitized patients [23]. The mechanism is largely unknown but may involve binding to Fc receptors on immune cells, inhibiting IgG production or inducing B cell apoptosis [35]. Our results suggest that IVIG inhibits IgG without reducing the number of B cells, although modest depletion of B cells may not be detected by our assay.

After rituximab, the changes in B cell repertoires were pronounced with significant decreases in the fraction of unique sequences, which is consistent with studies showing a long-lasting reduction in B cells after rituximab [36]. We also observed an increase in switched isotypes after rituximab, with more than half of candidates’ repertoires containing 90% switched molecules. The mutation frequency of the switched molecules increased slightly after rituximab, but the mutation frequency in the remaining non-switched molecules increased substantially to levels comparable to the switched molecules. Our study was not large enough to determine the precise temporal dynamics of the repertoire change in each candidate following rituximab therapy. Other work reported that B cell depletion persists for more than 180 days following treatment [36]. All but one of the post-rituximab samples was collected within 60 days following therapy with no significant differences in class-switched abundance or mutation frequency between samples collected within this interval. If the peak response following rituximab occurs after 60 days or later, our findings, which include many samples collected in the first 30 days, may underestimate the magnitude of the response. Larger studies are required to further characterize the individual variability in response after rituximab.

Rituximab, a chimeric CD20 monoclonal antibody, is postulated to decrease the production of HLA antibodies through targeting memory and naïve B cells without having any known effect on plasmablasts or plasma B cells, which do not express CD20 [36, 37]. Our findings may suggest an increase or a persistence of memory B cells or plasmablasts after rituximab. These results are consistent with other work showing that rituximab can effectively decrease memory B cells, but the relative fraction of memory B cells in both peripheral blood and lymph nodes can also increase [36, 38]. In addition, studies show that rituximab preferentially targets unmutated naïve B cells over class-switched mutated cells of the IgG and IgA isotypes [36–38]. However, we did not find any significant differences in B cell repertoires between responders and non-responders after rituximab therapy. Similar work in a B cell mediated neuropathy was able to identify differences in abundance, mutation frequencies and persistent clones in B cell repertoires between patients that did and did not respond to rituximab [39].

In the two candidates who received bortezomib close to rituximab, one showed changes similar to the other candidates receiving rituximab, whereas the other candidate showed little change. Bortezomib, a proteasome inhibitor, results in apoptosis of plasma cells although the effect on memory B cells is unknown [24, 40]. In this limited analysis, we were unable to detect any additional effect of bortezomib in these two candidates, but larger studies are needed to confirm these preliminary findings.

Kidney transplantation and the accompanying immunosuppression resulted in the opposite effect on the B cell repertoires as rituximab. We interpret the increasing fraction of unique sequences, reduction in the abundance of switched isotypes and strong decrease in mutation frequency within non-switched isotypes as representing the repopulation of naïve B cells after transplantation and immunosuppression. It is possible that we are underestimating the magnitude of these effects since the four candidates that did not receive rituximab experienced larger changes than those that received rituximab. The sampling interval does not appear to affect the change in class-switched mutation frequency following transplantation with the exception of two post-transplant samples, showing a small increase in class-switched isotype abundance, which were collected approximately 10 days earlier than the others. At the time of transplantation, all candidates receive ATG, which depletes T cells and leads to apoptosis of many B cell lineages without a measurable effect on peripheral B cell counts [30, 41, 42]. Reducing T cells may indirectly lower the mutation frequency since helper T cells are required for activating naïve B cells and triggering affinity maturation. Although MMF inhibits B cell proliferation with therapeutic uses in autoimmune disease, the exact effect on different B cell populations after transplantation is unknown [43].

Strengths of this study include the single study design that allows uniform and longitudinal desensitization protocols and sample processing as well as prospective and consistent HLA antibody monitoring. Another strength is our assay, which can detect ample B cell transcripts even after depletion by rituximab and can measure isotype classes after IVIG infusions, which interfere in serum immunoglobulin measurements. The assay implements bulk repertoire sequencing from total RNA, which yields isotype information and a robust signal due to multiple RNA copies per cell. Variability in RNA expression levels between cells, however, has the negative effect of complicating estimates of cellular clonality. Using total RNA simplifies the workflow, is faster, and reduces cost compared with sorted cells, but some cell types are not distinguished, such as plasmablasts and memory B cells. Both of these cell types are class-switched with elevated mutation frequencies, but plasmablasts secrete antibodies whereas memory B cells do not.

## Conclusions

This study demonstrates the application of high throughput sequencing technology to longitudinally monitor B cell repertoires in a challenging clinical setting of highly HLA-sensitized kidney transplant candidates. We demonstrate that B cell repertoires are dramatically affected by rituximab and transplantation with no observed changes after IVIG. We detected small differences at baseline that may distinguish responders from non-responders, and larger, prospective studies are required to validate these findings. This work builds on our multivariate analysis model reporting that differences in immune and gene profiles may help differentiate candidates that respond to desensitization therapy [29].

## Additional files

**Additional file 1: Table S1.** Candidate demographics, cPRA level and clinical summary. **Table S2.** Detailed sample information. **Table S3.** Primer sequences. **Table S4.** Mutation frequency correction factors.

**Additional file 2.** B cell cDNA sequencing library. (1) Full length template molecules of B cell mRNA contain 5′ untranslated region (U), leader sequence (L), V gene (V), D gene (D), J gene (J) and constant regions (C). (2) Reverse transcription from isotype-specific primers (IgG, IgA, IgM, IgD, IgE) into cDNA is performed in the presence of a generic template switching oligo that contains a 9-mer of randomly synthesized bases and a partial Illumina sequencing adapter. (3) 30 cycles of PCR amplification are performed with three primers: a partial P5 Illumina adapter containing 4 random bases for sequencing diversity on the 5′ end and on the 3′ end a partial P7 Illumina adapter containing a 6 base sample index (denoted “XXXXXX”) and a shorter primer without the index for more efficient amplification. (4) 6 cycles of PCR are performed to complete the P5 Illumina adapter including the short P7 primer on the 3′ end. (5) The first 150 bp read determines the isotype, J gene and CDR3 region and the second read includes the 5′UTR, leader sequence and 30–80 bp into the V region.

**Additional file 3.** Isotype abundances and mutation frequencies. **a. and b.** Results before and after IVIG. **c. and d.** Results before and after rituximab. **e. and f.** Results before and after transplantation.

**Additional file 4.** Change in class-switched isotype abundance and mutation frequency following rituximab and transplantation versus sampling interval. **a and b.** There were no significant differences between samples collected before or after 40 days (*dashed line*) following rituximab for either the change in class-switched isotype abundance (a) or mutation frequency (b). **c.** After transplantation, two samples with an increase in class-switched isotype fraction but otherwise no significant effects due to sampling interval. **d.** The change in class-switched mutation frequency following transplantation is not affected by sampling interval.

## Abbreviations

CDR3: complementarity-determining region 3
cPRA: calculated panel reactive antibody
ESRD: end-stage renal disease
HLA: human leukocyte antigen
IVIG: intravenous immunoglobulin
