# Supplementary figures and images for: B cell repertoires in HLA-sensitized kidney transplant candidates undergoing desensitization therapy
Source: J Transl Med. 2017 Jan 13;15:9. doi: 10.1186/s12967-017-1118-7 (PMC5237299; doi:10.1186/s12967-017-1118-7)

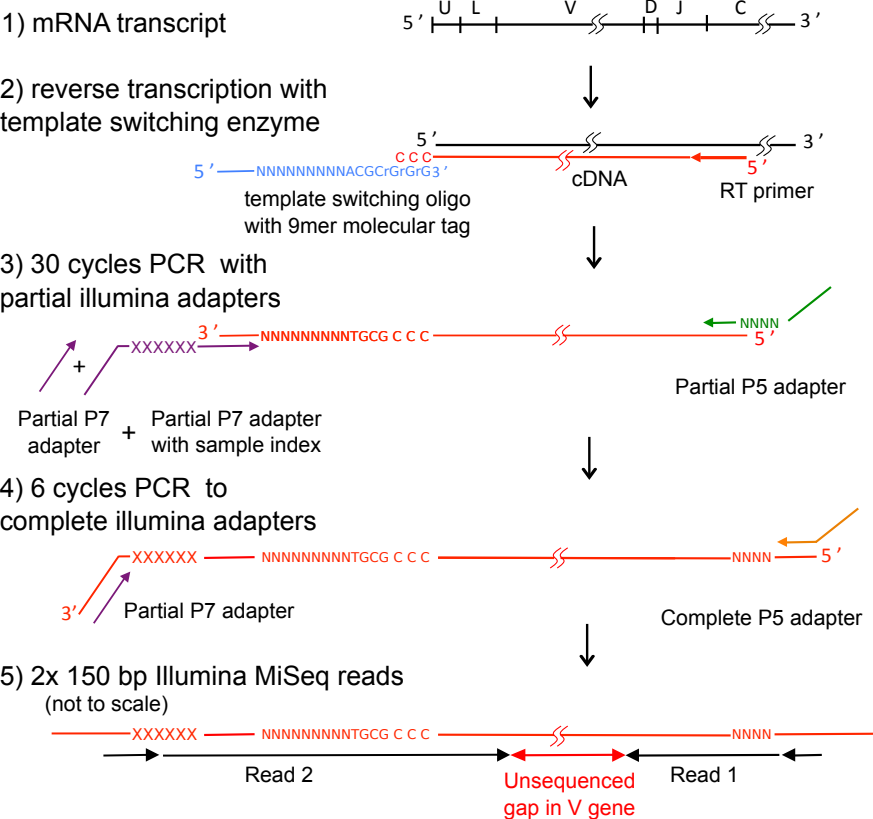

Supplement: Supplementary file 2 — Additional file 2. B cell cDNA sequencing library. (1) Full length template molecules of B cell mRNA contain 5′ untranslated region (U), leader sequence (L), V gene (V), D gene (D), J gene (J) and constant regions (C). (2) Reverse transcription from isotype-specific primers (IgG, IgA, IgM, IgD, IgE) into cDNA is performed in the presence of a generic template switching oligo that contains a 9-mer of randomly synthesized bases and a partial Illumina sequencing adapter. (3) 30 cycles of PCR amplification are performed with three primers: a partial P5 Illumina adapter containing 4 random bases for sequencing diversity on the 5′ end and on the 3′ end a partial P7 Illumina adapter containing a 6 base sample index (denoted “XXXXXX”) and a shorter primer without the index for more efficient amplification. (4) 6 cycles of PCR are performed to complete the P5 Illumina adapter including the short P7 primer on the 3′ end. (5) The first 150 bp read determines the isotype, J gene and CDR3 region and the second read includes the 5′UTR, leader sequence and 30–80 bp into the V region. [file 12967_2017_1118_MOESM2_ESM.pdf]

Control = ○  
 Non-Responder = △  
 Responder = □

PreT = Pre-Treatment  
 PreR = Pre-Rituximab

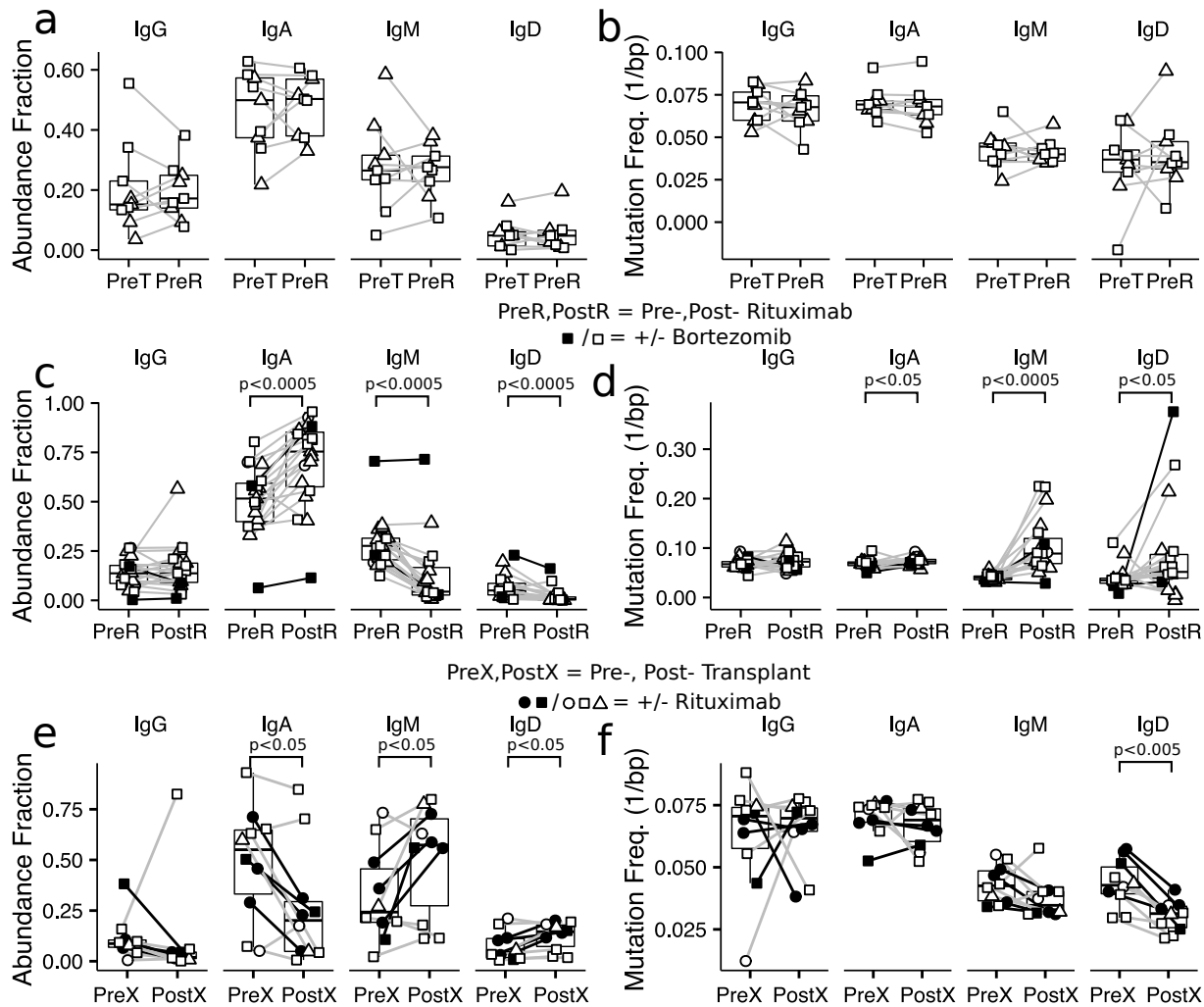

Supplement: Supplementary file 3 — Additional file 3. Isotype abundances and mutation frequencies. a. and b. Results before and after IVIG. c. and d. Results before and after rituximab. e. and f. Results before and after transplantation. [file 12967_2017_1118_MOESM3_ESM.pdf]

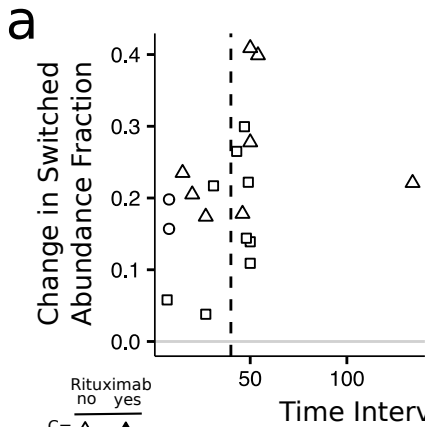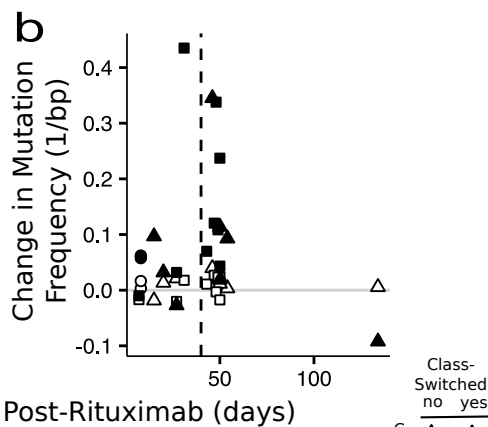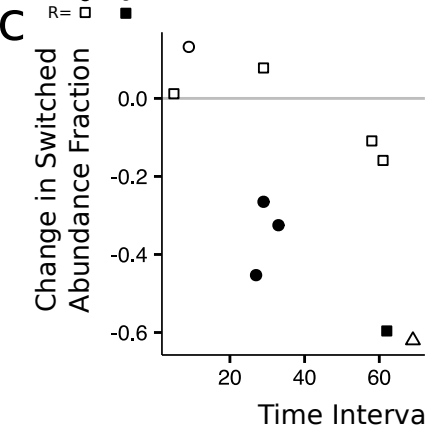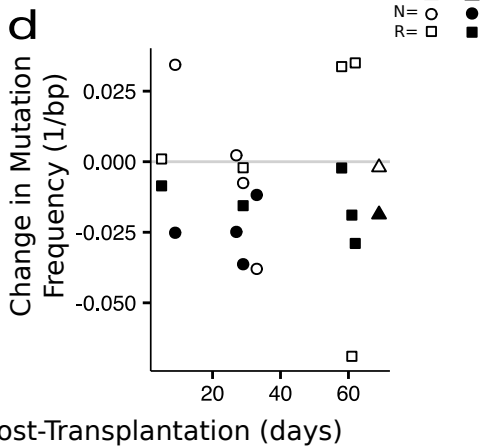

Supplement: Supplementary file 4 — Additional file 4. Change in class-switched isotype abundance and mutation frequency following rituximab and transplantation versus sampling interval. a and b. There were no significant differences between samples collected before or after 40 days (dashed line) following rituximab for either the change in class-switched isotype abundance (a) or mutation frequency (b). c. After transplantation, two samples with an increase in class-switched isotype fraction but otherwise no significant effects due to sampling interval. d. The change in class-switched mutation frequency following transplantation is not affected by sampling interval. [file 12967_2017_1118_MOESM4_ESM.pdf]
